# Delayed Onset Scleromalacia and Conjunctival Bleb Formation Following Intraoperative Mitomycin C Application during Conjunctival Melanoma Excision

**DOI:** 10.3390/vision4020024

**Published:** 2020-05-06

**Authors:** Syed Naqib Ahmed, Syed Mohammed Shahid, Mayank A. Nanavaty

**Affiliations:** 1 Eastbourne District General Hospital, Kings Drive, Eastbourne BN21 2UD, UK; syed.ahmed15@nhs.net; 2 Moorfield Eye Hospital, City Road, London EC1V 2PD, UK; ss4562@doctors.org.uk; 3 Sussex Eye Hospital, Brighton & Sussex University Hospitals NHS Trust, Eastern Road, Brighton BN2 5BF, UK; 4 Brighton & Sussex Medical School, University of Sussex, Falmer, Brighton BN1 9PX, UK

**Keywords:** conjunctival melanoma, mitomycin C, scleritis, scleromalacia

## Abstract

Purpose: To present a case of delayed onset scleromalacia and conjunctival bleb formation one year after conjunctival melanoma excision with intraoperative use of mitomycin-C (MMC) and double-freeze-thaw technique. Methods: Case report. Results: A 69-year-old woman had a conjunctival melanoma excised by the ‘no touch technique’ with intraoperative application of 0.02% MMC for 3 min on bare sclera, freeze-thaw cryotherapy and amniotic membrane transplant performed elsewhere. Three months later, she presented to us with a red, sore and painful right eye. Examination revealed severe scleritis. She was treated with lubricants and oral steroids for 6 weeks. She settled well with no recurrence of melanoma. At one year, she developed scleromalacia and conjunctival blebs in the inferonasal quadrant of right eye. She remains under closer observation as she is at high risk of perforation. Conclusion: Caution should be exercised with intraoperative use of MMC on bare sclera during excision and cryotherapy of conjunctival melanoma. As published in the literature, when using MMC, it is recommended to use the lowest dose topically in the form of eye drops in the postoperative period for the shortest time to avoid any sight-threatening complications.

## 1. Introduction

MMC is an antibiotic with anti-neoplastic activity that has previously been used intravenously to treat a variety of systemic tumors. The chemotherapeutic drug is isolated from the soil bacterium *Streptomyces caespitosus*. It is currently also used topically in bladder tumor resection, and intra-peritoneal tumors. Within Ophthalmology, topical use was initially advocated to prevent recurrence in pterygium surgery. More recently, clinical uses in eye surgery have gained popularity and include prevention of haze in refractive surgery, prevention of scarring in glaucoma filtering surgery, use in squint surgery, dacryocystorhinostomy, allergic conjunctivitis and ocular surface tumors’.

We describe a rare complication of delayed onset scleromalacia and conjunctival bleb formation post-excision of conjunctival melanoma with intraoperative application of MMC on bare sclera, double-freeze-thaw cryotherapy and amniotic membrane transplant.

## 2. Case Report

A 69-year old lady underwent an excision of a suspicious dark pigmented conjunctival lesion in the inferonasal region of her right eye in June 2017 elsewhere. This surgery involved conjunctival excision biopsy with 3mm borders and a ‘no-touch technique’ [1] with double-freeze-thaw cryotherapy and topical mitomycin-C (MMC) 0.02% for three minutes, along with reconstruction with an amniotic membrane graft. Subsequent pathology report concluded the lesion to represent a melanoma.

**Figure 1:**
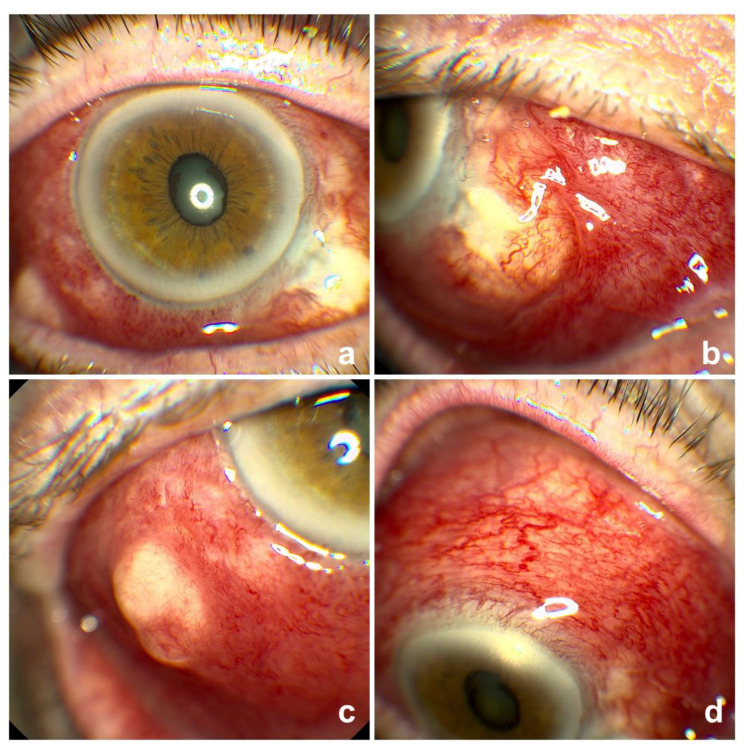
Right with scleritis at presentation 3 months after the procedure. (**a**) Axial view; (**b**) Inferonasal part of the sclera (primary site of the procedure); (**c**) Suspicious lesion which was biopsied and found to be just an inflammatory mass at this presentation; (**d**) Extensive scleritis.

In August 2017 (3 months later), she presented to the eye casualty at the Sussex Eye Hospital, Brighton and Sussex University Hospitals NHS trust, Brighton, United Kingdom with red, sore and itchy right eye for five weeks (Figure 1a–d). At the time of presentation to our department, the patient was on Ofloxacin and Dexamethasone eye drops four times a day to the right eye.

She had no past ocular history. Her past medical history included hypertension, pulmonary embolism and diverticulitis. She took regular amlodipine, losartan and apixaban with no relevant family history. The patient worked as a farmer, which predisposed her to ultraviolet light exposure.

On examination, visual acuity was 20/200 (corrected 20/60) in the right eye, and 20/10 in the left eye. The left eye was structurally normal. Intraocular pressures were normal. Fundus examination was normal. Ultrasound B-scan and a gentle ultrasound biomicroscopy showed no intraocular mass lesion.

**Figure 2:**
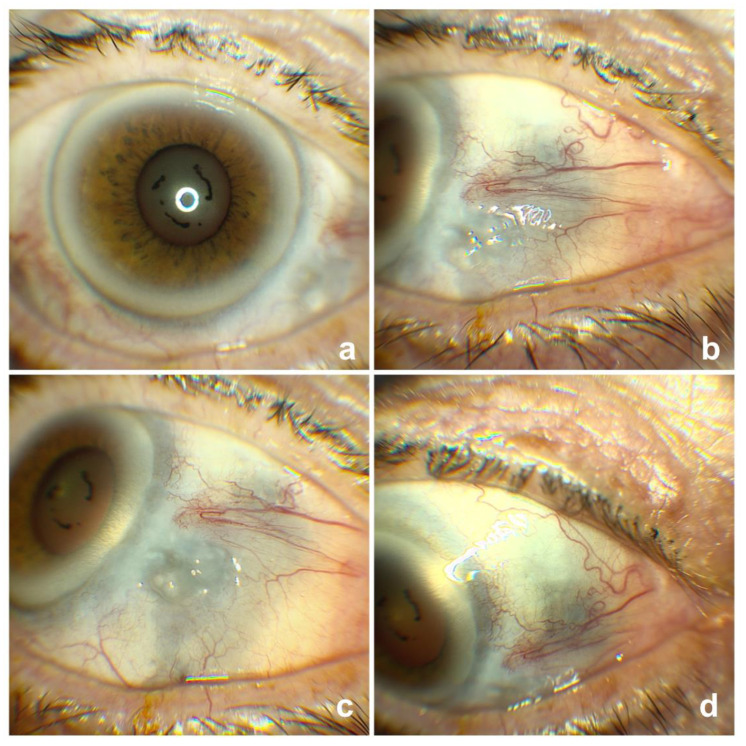
Right eye at 1 year follow up (**a**) Axial view; (**b**) Inferonasal part of the sclera with scleromalacia (primary site of the procedure); (**c**) Conjunctival blebs due to significant scleral thinning; (**d**) Another view of scleromalacia on nasal side.

She was diagnosed with severe scleritis (Figure 1a–d). There was a suspicious elevated area inferotemporally (Figure 1c). This was reported to be inflammatory in nature (rather than neoplastic) upon a shave excision biopsy on the day of presentation. We treated her with intensive preservative free lubricants and oral Prednisolone 60 mg tapering over 6 weeks with antacids cover. Following this treatment, she settled with no evidence of thinning or recurrence of melanoma. A year later, she was noted to have a large patch of scleromalacia with significant scleral thinning and conjunctival blebs in the inferonasal quadrant of the right eyeball (Figure 2a–d). She remains under 6 weekly observations in the clinic to look out for signs of perforation.

## 3. Discussion

For ocular tumors, such as those originating from melanocytes, surgery alone would, historically, have a recurrence rate of 50% [2]. Topical drugs are therefore used as adjuvant therapy postoperatively. MMC has an anti-proliferative effect by selectively inhibiting DNA synthesis, RNA transcription and protein synthesis. The mechanism of action of DNA inhibition is achieved by cross-linking at the N position of adenine and 06, and N position of guanine bases [3]. The dose used for topical treatment is usually 0.02–0.04% MMC. The typical treatment regime used is 0.04% MMC eye drops four times a day for three weeks [4]. Majority of the published evidence suggest the use of MMC drops postoperatively rather than intraoperative application on bare sclera for conjunctival melanocytic lesions. With regards to the methods of application of MMC, the time-tested route is via a sponge soaked in it [5]. This sponge is applied to the sub conjunctival space. Both the concentration of the drug used, and the duration of exposure can be altered, depending on the risk of failure. A subconjunctival injection of MMC instead of these sponges is recently being studied as an alternative in glaucoma surgery with some evidence in glaucoma surgery that the subconjunctival injection method of application of MMC is associated with superior surgical outcomes and no increase in complications [6,7]. In sponge application, the surface area of cut pieces of surgical sponges is very variable and a study found that the quantities of MMC contained in sponges prepared for glaucoma surgery differed for a given surgeon and between surgeons [8]. The estimated actual dose delivered in a sponge soaked with MMC 0.2 mg/mL varied between 1.9 and 17.3 μg [8]. With this unpredictable sponge dosing, surgeons run the risk of overdosing MMC and this could be the possible reason of our case. Moreover, irrigation is used after delivery of MMC which only have an effect at reducing MMC concentrations in the superficial scleral layers, with no effect on MMC concentrations in the deep scleral and sub-scleral layers [9]. However, it has to be noted that subconjunctival route of MMC application is not very popular in cases with ocular surface melanoma. ‘No touch technique’ is an established technique for removal of conjunctival melanocytic lesions and has shown reduced risk of recurrence due to seeding and metastasis [1,10]. as in our case.

A variety of complications have been documented with the use of MMC in ophthalmology. In the majority of cases, complication reports are seen in patients who have undergone pterygium excision. One case, for example, showed scleral dellen to be an early complication that can occur 1–12 days post-pterygium surgery [11]. Further reports on scleral complications include necrotizing scleritis, with or without scleromalacia, ulceration and calcification. These complications were also limited to pterygium excision with adjunctive MMC [12,13,14]. Necrotizing anterior scleritis is also reported after regional conjunctivectomy with postoperative topical MMC [15], which may be due to epithelial breakdown and fibroblast damage owing to the use of MMC, as well as vascular compromise owing to extensive conjunctivectomy [16,17]. In a recent study by Ji et al. [15], the average time of presentation with necrotizing scleritis following cosmetic conjunctivectomy in Korea were 51 months. However, in this study [15] they used topical MMC drops postoperatively. Our patient presented with scleritis earlier (3 months later) and scleromalacia with conjunctival blebs 12 months and this could be due to bare sclera application of MMC 0.02% for 3 min intraoperatively. Iatrogenic deprivation of vessels causes localized ischemia and facilitates scleral necrosis [15]. Furthermore, epithelial defects and bare sclera contribute to prolonged scleral exposure to antigens, which may elicit an initial immune reaction [18,19,20,21]. In addition, MMC decreases conjunctival goblet cell density, resulting in insufficient mucin secretion by the remaining conjunctiva [22]. This alteration in tear film may excessively expose the underlying sclera to desiccating stress.

On the other hand, studies assessing management of ocular surface squamous neoplasia found complications like lid allergy, corneal epithelial defect, epiphora secondary to punctal stenosis, and ectropion secondary to cicatricial changes to the lower lid unlike our case. However, these studies showed no long-term complications unlike our case [23]. Additionally, a long-term review of 58 patients who were treated with topical MMC for ocular surface neoplasia of different types found short-term complications such as allergy to be common. The only significant long-term complication found in seven of the fifty-eight patients was limbal stem cell deficiency [24].

Our report identifies delayed scleromalacia with conjunctival blebs to be a further complication found in patient who have been treated with primary excision for melanoma with MMC therapy intraoperatively. The case therefore highlights the importance of scleral thinning as a serious complication following intraoperative use of MMC in addition to cryotherapy and amniotic membrane transplant in patients with conjunctival melanoma. Caution should be implemented whenever MMC is considered as an intraoperative adjunct for bare sclera application compared to postoperative topical therapy with milder concentrations.
